# How predictive medicine leads to solidarity gaps in health

**DOI:** 10.1038/s41746-025-01497-2

**Published:** 2025-02-18

**Authors:** Matthias Braun

**Affiliations:** Department of Social Ethics, University of Bonn, Bonn, Germany

**Keywords:** Ethics, Society

## Abstract

The current shift in healthcare towards AI-driven P4 medicine challenges practices of solidarity, with implications for EU health policy.

In recent years, there has been a shift from personalised medicine to predictive medicine. One concept that illustrates this shift is the concept of highly personalised, preventive, participatory, and predictive medicine (known as P4 medicine). A central goal of P4 medicine is no longer just to find the best individual treatment option, as precision medicine suggests. Rather, it is about predicting the onset of disease as accurately as possible, forecasting the course of disease, and adapting behaviour in response to the predictions. P4 medicine has gained significant traction in recent years and has become even more important with the integration of AI^1^.

P4 medicine has major implications for access to healthcare and especially for practices of solidarity as a fundamental social value. So far, practices of solidarity have not played a central role in the major debates on the regulation and governance of precision medicine, particularly on the integration of AI: In the debates on the AI Act (where solidarity is not mentioned in the current proposal), the European Data Act, or the European Health Data Space (EHDS) regarding the use of primary and secondary health data, the concept of solidarity is notably absent. In practice, however, precision medicine threatens to fragment solidarity, both because of the greater variation of costs and because of the priority given to treating diseases such as cancer over other life-threatening diseases^2,3^.

In this article, I will analyse the implications of P4 medicine on solidarity and discuss two major challenges for solidarity, which I will call the two solidarity gaps caused by the predictive dimension of P4 medicine.

## The case of P4 medicine

The primary objective of P4 medicine is shifting from merely identifying the optimal treatment for individuals to focusing on accurate disease onset, charting the disease’s trajectory, and modifying behaviours based on predictive insights^4^. The aim is to enable the best diagnosis, behavioural adjustments, or suitable therapies, as in the case of digital twins. Digital twins are used to test the most suitable medication or surgical procedure and to predict the onset of diseases such as dementia^5^, stroke^6^ or certain cancers^7^.

With this agenda, P4 medicine is now bringing about a further change: there is a decoupling of symptoms (i.e. a form of pain or harm) and the need for health services. If a prediction, based on an analysis or simulation of physical characteristics, predicts that a particular person will suffer a stroke in two weeks or develop dementia in six months with a probability of 70 per cent, in many cases there are no symptoms and often no restrictions at the moment of the prediction^8^. This raises two important questions: On a personal level, to what significance is the prediction meaningful for the person concerned? On an institutional level, to what extent can and should a prediction form of P4 medicine offer access to health services?

P4 medicine is not only a debate about the future of medicine but also about the trajectories of shaping the future of society in general. Shaping our future in such a way that it is fair and inclusive for as many people as possible, while at the same time preserving, establishing, and creating stable institutions in which these futures can be debated and shaped, is one of the major tasks that societies are currently facing. Such an entangling of existing power imbalances is also one of the central ideas within the European Union: Shaping the future should, and must, be value-based to ensure a just and common basis for action, whether it is the implementation of the United Nations 2030 Agenda on sustainability issues, the strategy for European digital sovereignty or declarations such as the Berlin Declaration on value-based governance strategies^9,10^.

### P4 medicine and its impact on solidarity

At the same, the predictive and preventive focus of P4 medicine is not only fostering a debate about desirable futures but is changing the meaning and function of certain values, in particular solidarity^11–15^. Solidarity can be defined as a shared commitment to accepting costs to support others who are recognisably similar in some respect. In this sense, solidarity denotes a *practice* of being mean to someone. Taking into account that solidarity has three tiers (inter-personal, communal, and institutional), access to healthcare mainly addresses the institutional tier of solidarity. The concept of solidarity focuses on collective support and mutual aid, especially in health governance and the broader societal context. Solidarity involves actively engaging in collective efforts and concerns, rising significantly where individuals or groups face injustice. Institutional practices of solidarity (i.e., making oneself common with the affairs of others) emerge precisely where people have specific experiences of injustice^16–18^.

In this sense, solidarity has an ambivalent role between *creating* something (like a social bond) and *indicating* experiences of injustices: On the one hand, it refers to a bundle of social practices that are essential for creating something like a social bond^19^. Legal systems, democratic structures, and debates about values all require a precondition that they cannot create themselves: A fundamental notion of a collectively shared common narrative and experience. For example, the European Union intends to shape futures based on core values, placing solidarity alongside trust at its core. In the context of health, this means that people experience themselves as physical and vulnerable beings and show solidarity with others’ experiences of illness. Institutions such as healthcare systems are built on the widely accepted (both legally and morally) traditional notion that everyone faces uncertainty regarding if, to what degree, and when they need access to healthcare. The possibilities of AI-driven P4 medicine are transforming this uncertainty by enabeling far more precise predictions about if, to what degree, and when someone will need access to healthcare.

On the other hand, solidarity may be understood as an indicator of experiences of injustice and the potential loss of trust in institutions. Practices of solidarity (which generally refer to making oneself common with the affairs of others) emerge – and have their particular functional significance for health governance – precisely where people have specific experiences of injustice^17,18^. Not infrequently, such experiences of injustice stem from structural modes of organisation and are associated with experiences of loss of trust in institutions^20–23^. To put it more pointedly, a high level of solidarity practices could indicate a low level of trust in the resilience of institutions. At the same time, however, recognising acts of solidarity can create new bonds of trust, whether through shared experiences or the appreciation of commonalities. This is particularly true when attempts are made to translate trust into specific formulas and criteria aimed at establishing rules for healthcare and the use of a specific technology. This is not a bad idea in and of itself, if one does not succumb to the belief that these criteria can guarantee trust.

## Solidarity gaps – and how to fix them

There is currently a tension between the ideals of solidarity and the realities of its implementation in governance, particularly with the rise of P4 medicine and AI.

With a greater focus on predicting disease events before they occur, there are not only shifts but also tensions regarding practices of solidarity. To understand and address these shifts in EU regulation, I discuss two solidarity gaps (referring to debates on potential responsibility gaps)^24–26^ as focal points where action is needed. The first gap addresses the question of how to *attribute meaning* when dealing with the given predictions. This is an important issue as with the experience of pain or harm there is already a clear notion that harm should be avoided. This is not necessarily the case while using predictions as one basic tool to regulate access to healthcare. The second one focuses on the shift that predictive medicine will cause in the regulation of access to public healthcare. Both gaps address the tensions of maintaining community ties amidst technological advancements and how institutions can foster trust in dealing with them. These two gaps in solidarity emerge with the shift towards prediction, as there is no longer a mandatory link between pain or harm and a solidaric sharing of healthcare costs. It is precisely this disconnection of the experience of symptoms (in the sense of pain or harm) that puts the established institutional routines of practising solidarity under pressure. This is a very concrete issue for organising access to healthcare as well as for deliberating and justifying the rationale of rationing resources^7^.

The first gap arises when new common practices are required for attributing of meaning. This point has been neglected in previous debates on EU regulation and governance of AI. In most healthcare systems, an entitlement to healthcare exists when symptoms of illness occur and a diagnosis is confirmed. There are some exceptions, such as in the case of high blood pressure or certain risk profiles. However, in principle, the value of solidarity in healthcare contexts is based on mutual reciprocity in healthcare cost-sharing. The crucial point is that predictive medicine changes the reason and basis for showing solidarity and sharing costs. In current systems, the occurrence of a disease, the personal matter of how an individual attributes meaning to a symptom, and the societal negotiation of what should be recognised as a sickness are linked to the tangible experience of bodily incapacity. This changes with the prediction of a possible future health event and a recommendation given, for example, by a digital twin to adjust behaviour. It also fundamentally changes the question of when public healthcare systems act in solidarity. No longer is it primarily the experience of suffering and pain that counts as decisive, but rather the narratives of a certain individual or the social interpretation of a given prediction.

At this point, the debate on the governance of predictive medicine needs to be broadened. Not only are technical artifacts and systems relevant in a narrow sense, but so too are the techniques used to attribute meaning to predictions. More specifically, this means that debates about the responsible use of predictive medicine must not solely focus on hard and soft law, but also require the ability to narrate and interpret – and to ground these narratives and interpretations as an essential part of EU governance approaches.

A second solidarity gap occurs in the handling of the predictions received in a public healthcare system. The purpose of generating a prediction will not always be readily apparent. But a person must have the right (and the means to assert this right) to opt out receiving a particular health warning, or even a particular probability of risk. This applies both to the question of which predictions a person receives and to how the prediction is dealt with in healthcare institutions. It would represent a clear break with the EU’s solidarity-based health policy if, for example, predictions about future health events and information about the nudges a person has received from a digital twin were shared with insurance companies. It would also run counter to the understanding that human life is fundamentally vulnerable, and deny the rationale for treating people differently, such as those with disabilities or a particular desire to engage in high-risk sports. Shifting the burden of responsibility to the individual model flies in the face of what we know about structural injustice, marginalization, and discrimination. It implies a willingness to accept medium- and long-term disadvantages for possible short-term cost reductions.

To address the second gap, the patient’s right to control the use of their health outcome predictions must be an essential facet of a solidarity-based EU governance approach^27^. At the same time, institutional structures are needed to enable and support this right. It is essential to ensure interoperability between individual regulatory and governance approaches. In addition, routines must be established to assess whether interoperability is functioning effectively. This is currently not the case as for example, the debates on the Medical Devices Regulation, the AI Act, and the European Health Data Hub are taking place in separate silos^28^.

Further, such a focus on interoperability needs to be supported by urging healthcare institutions to define where and when they should be blind to specific predictions^29^. For example, in the clinical healthcare sector, predictions and health recommendations generated by AI medical technologies may be useful for more proactive and early interventionist approaches to patient care. But what information, if any, about these AI-generated predictive outcomes and data should clinics share with health insurers? Defining the parameters for how health information is handled and shared with such companies is paramount. If it is true that all data is health data, it will not be possible or reasonable to prohibit predictions^30^. AI-driven medicine will play a central role in future healthcare. This may not be a bad thing if EU policy and regulation start now to establish solidarity-based governance structures to push institutions to define which predictions can and should be used.
